# Beyond the Scalpel: A Tapestry of Surgical Safety, Precision, and Patient Prosperity

**DOI:** 10.7759/cureus.50316

**Published:** 2023-12-11

**Authors:** Frank Ansah Owusu, Herra Javed, Ayesha Saleem, Jagjeet Singh, Giustino Varrassi, Syed S Raza, Raja Ram

**Affiliations:** 1 Medicine, Stavropol State Medical University, Stavropol, RUS; 2 Surgery, Shifa College of Medicine, Islamabad, PAK; 3 General Surgery, Hayatabad Medical Complex (HMC), Peshawar, PAK; 4 Internal Medicine, Lahore General Hospital, Lahore, PAK; 5 Pain Medicine, Paolo Procacci Foundation, Rome, ITA; 6 Physiology, Gajju Khan Medical College, Swabi, PAK; 7 Physiology, Khyber Medical College/Teaching Hospital, Peshawar, PAK; 8 Robert and Suzanne Tomsich Department of Cardiothoracic Surgery, Cleveland Clinic Florida, Peshawar, PAK; 9 Physiology, Gandhara University, Peshawar, PAK; 10 Medicine, MedStar Washington Hospital Center, Washington, USA

**Keywords:** general surgery, precision, safety, surgery, surgical, scalpel

## Abstract

In modern surgical practice, the focus extends beyond simply making and closing incisions. We aim to investigate the various complex aspects that redefine the criteria for achieving effective surgical outcomes. This narrative combines current knowledge, integrating practical experiences and academic viewpoints to comprehend the changing field of surgical care thoroughly. The tapestry explores the detailed aspects of surgical safety, examining the most recent progress in protocols, technology, and team dynamics that strive to reduce procedural risks. Examining precision in surgery, this narrative goes beyond conventional limits to explore the incorporation of advanced technologies, such as robotics and navigational systems. The complex interplay between the surgeon's proficiency and these technology aids is crucial in attaining unparalleled accuracy and favorable patient results. The focal point of this investigation is the patient's well-being, encompassing postoperative care, rehabilitation, and long-term health. Actual accounts from surgical procedures highlight the significant influence of comprehensive patient-centered methods, emphasizing the crucial need for empathy, communication, and individualized care plans in promoting healing and adaptability. As we explore this complex situation, the combination of real-life stories and academic discussions creates a clear and detailed image of a surgical environment that goes far beyond the boundaries of the operating room. "Beyond the Scalpel" seeks to engage practitioners, scholars, and stakeholders in a conversation that redefines the criteria for surgical success. It aims to establish a new benchmark that combines safety, precision, and patient well-being, ultimately shaping the future of surgical practice.

## Introduction and background

The advancement of surgical practice within the complex fabric of medical progress is a tribute to humanity's unwavering dedication to achieving perfection in healthcare. Throughout decades, surgical techniques have evolved from basic procedures filled with danger to advanced treatments characterized by accuracy and security [1]. The story of surgical progress is marked by ingenuity, perseverance, and an unwavering dedication to improving patient results. Our initial focus is on the fundamental significance of surgical safety. Traditionally, the operating room has been characterized by uncertainty, where the distinction between success and failure was frequently precarious [2]. Over time, ensuring safety during surgical procedures has become fundamental, influencing the creation of norms, standards, and technology to reduce risks and consequences. The progress of surgical safety, ranging from aseptic procedures to sophisticated monitoring systems, reflects society's shared dedication to guaranteeing that the advantages of operation significantly surpass its potential risks [3]. The second theme emerges as we explore the complex terrain of accuracy in surgical procedures. With the rapid advancement of technology, the surgeon's toolset has also expanded to incorporate minimally invasive techniques, robotic aid, and real-time imaging. The rise of precision in surgical procedures has transformed it from a mere goal to a genuine achievement [4]. Surgeons are now able to maneuver through intricate anatomical structures with unmatched accuracy. With a focus on accuracy, we observe a transformation in surgical abilities, where previously intrusive techniques have been replaced with methods characterized by delicacy, effectiveness, and minimized patient harm [5].

The third focus of our investigation revolves around the primary objective of surgery - the well-being and success of the patient. In addition to promptly addressing medical concerns, surgery today aims to improve general wellness and enhance the quality of life [6]. Patient-centered treatment encompasses preoperative preparation, postoperative rehabilitation, and a comprehensive evaluation of the patient's physical, emotional, and social aspects, extending beyond the operating room [7]. The development of surgical practice is closely linked to the notion of patient well-being since achievement is currently evaluated not only in terms of survival but also in terms of restored functionality, enhanced aesthetics, and a resumption of a satisfying life [8].

As we strive for surgical excellence, it becomes clear that safety, precision, and patient well-being are closely connected. The importance of adopting a holistic approach to surgery lies in acknowledging the interdependence of these elements and integrating them into a comprehensive framework that goes beyond the limitations of individual procedures. A comprehensive approach recognizes that surgery is not just a single event but a process that starts when the patient interacts with the healthcare system and continues well beyond the operating room.

## Review

Surgical safety

Historical Context

The inception of surgical safety protocols can be traced back to the groundbreaking contributions of Joseph Lister throughout the 19th century. Lister's promotion of antiseptic procedures revolutionized the field of surgery by establishing the basis for aseptic techniques [9]. Over the following decades, safety precautions were improved and expanded in response to advancing microbiology and infection control knowledge. Currently, surgical safety is equivalent to a thorough system that covers the stages before, during, and after surgery [10]. Preoperative examinations encompass comprehensive evaluations of patients, categorization of risks, and the development of personalized treatment plans. During the surgery, following aseptic techniques strictly is crucial, utilizing sophisticated sterilization methods, and maintaining a vigilant approach to prevent infections. After surgery, robust monitoring and follow-up systems guarantee the ongoing health and welfare of the patient [11]. In the modern surgical environment, there is a strong emphasis on collaboration between specialists from different disciplines, such as surgery, nursing, and anesthesia. These professionals come together to form cohesive teams. This creative synergy exemplifies the shared dedication to the health and welfare of patients. Efficient team dynamics, characterized by effective communication and collaboration, are essential for smoothly implementing safety standards [12].

Concrete instances exemplify the profound influence of safety standards in various surgical situations. The value of extensive safety precautions is highlighted by cases where thorough preoperative assessments identified hidden health problems, preventing possible complications [13]. The practical implementation of intraoperative safety guidelines is demonstrated in instances where diligent infection control measures averted postoperative consequences. These case studies show the tangible influence of safety measures on patient well-being [14].

Exploring the Impact of Technology on Surgical Precision

The current account of surgery is closely linked with technology breakthroughs that redefine accuracy. Technology plays a crucial role in improving surgical precision through various advancements, all of which enhance surgical procedures. Technology enhances surgeons' ability to use modern imaging modalities and precision instruments [15]. The fusion of robotics and navigation systems is at the forefront of technical advancement in surgery. Robotics, with their complex dexterity and improved visualization, redefine the limits of surgical accuracy. Surgical navigation systems, utilizing real-time imagery, offer surgeons dynamic guidance, allowing for exceptional precision in navigating intricate anatomical structures [16].

Role of Accuracy in Achieving Favorable Surgical Results

The impact of precision in surgery on patient outcomes is substantial, extending beyond the mere integration of technology. Minimally invasive operations, aided by precise equipment and robotics, decrease injury, minimize scarring, and expedite recovery periods. Possessing the ability to navigate complex anatomical structures accurately leads to improved diagnoses, focused interventions, and favorable surgical results. Real-life examples of precision technologies demonstrate their ability to change many surgical settings significantly [17]. Case studies exemplify the efficacy of robotic-assisted procedures in minimizing scarring, diminishing postoperative pain, and expediting recovery durations. Navigation-assisted treatments, especially in intricate anatomies, have resulted in accurate tumor removals, reducing unintended harm and maximizing overall effectiveness. These case studies highlight the concrete advantages of precision technologies in influencing favorable patient results [18]. Beyond the Scalpel is not just a story but a monument to advancing surgical techniques. This tapestry combines traditional safety standards with modern breakthroughs in precise technology, creating a tale that goes beyond the boundaries of the operating room. The combination of safety, accuracy, and patient-centered care is becoming a model for successful surgical outcomes, with potential benefits extending beyond the immediate procedure [19]. This tapestry acts as a guide for surgeons as they negotiate the intricate aspects of modern surgery. It directs them toward a future where safety, precision, and the well-being of patients are intertwined, shaping the story of surgical greatness.

Patient prosperity

In surgery, patient well-being encompasses more than just the immediate cure of medical problems. It involves a comprehensive strategy prioritizing the individual's health and happiness. The core of this fundamental change is the notion of patient-centric care. This philosophy prioritizes the patient as the central focus of the healthcare experience [20]. In surgery, patient-centric care goes beyond being a trendy term. Instead, it represents a revolutionary strategy that acknowledges the distinct requirements, principles, and choices of every person having a surgical procedure. The implementation of personalized treatment plans, which involve adapting medical interventions to each patient's individual qualities and circumstances, has a tremendous impact on patient outcomes [21]. The effect of individualized care plans on patient outcomes is manifold, spanning not only the immediate postoperative period but also long-term healing and quality of life. With the growing adoption of precision medicine in surgical practices, treatment strategies are customized according to genetic, environmental, and lifestyle factors [22]. This benefits patients during surgery and brings about an era where patient well-being is closely tied to personalized healthcare interventions.

Empathy and excellent communication are crucial in promoting patient well-being in the surgical setting. The compassionate surgeon recognizes that the patient is not just a collection of medical records but a person struggling with emotions, such as fear, uncertainty, and the inherent vulnerability that comes with undergoing surgery [22]. By engaging in sympathetic communication, surgeons can form a therapeutic relationship with patients, which helps reduce anxiety and enables patients to engage in their healthcare journey actively. Recognizing the significance of the interpersonal bond in healthcare, surgeons prioritizing empathy and communication substantially contribute to their patient's psychological and emotional welfare, ultimately improving their overall well-being [23]. Treatment strategies and concerns for long-term well-being following surgery are essential elements of patient-centered treatment. Rehabilitation extends beyond the physical recuperation process, incorporating psychological and social factors to restore normal functioning. Postoperative rehabilitation encompasses physical therapy, counseling, and support groups, acknowledging the enduring effects of surgery that extend beyond the initial recovery phase [24]. Rehabilitation treatments enhance long-term well-being by addressing various aspects of a patient's experience, fostering resilience and adaptation in the face of postoperative difficulties.

Real-life stories exemplify effective patient-centered practices in the surgical setting. These accounts offer a look into the lives of patients who experienced personalized care, sympathetic communication, and comprehensive rehabilitation during their surgical journeys [25]. By sharing these narratives, we acquire a deeper understanding of the concrete effects of patient-centered care on actual individuals, surpassing mere statistical results to recognize the significant transformation that occurs when the healthcare system adopts the principles of personalization, compassion, and sustained welfare [26]. Patient prosperity in surgery extends beyond the boundaries of medical operations, highlighting the significance of patient-centered care, tailored interventions, compassionate communication, and thorough rehabilitation. Adopting these principles in surgical practices improves both short-term postoperative results and long-term well-being of patients [27]. This ensures that surgical excellence is achieved with elements of compassion, understanding, and dedication to the success of each individual.

Interconnected threads

The relationship between surgical safety, precision, and patient well-being is intricate and interconnected in healthcare. A comprehensive approach is the foundation of overall surgical success as these threads intertwine. This narrative examines the mutually beneficial link between these interwoven parts, investigating how improvements in safety and precision not only reduce risks but also directly improve patient outcomes [28]. The advancement of surgical safety has been characterized by careful attention, creativity, and dedication to reducing risks. In the past, surgical procedures were characterized by uncertainties and frequently posed life-threatening complications. Nevertheless, with the advancement of safety standards, aseptic techniques, and monitoring systems, the operating room has changed significantly, becoming a highly regulated environment where the delicate equilibrium between risk and benefit is meticulously adjusted [29]. The importance of surgical safety extends beyond preventing immediate risks. It plays a crucial role in establishing the groundwork for a patient's path to recovery and well-being [30].

The field of surgical practice has been revolutionized by technological breakthroughs, leading to a significant improvement in precision. Precision has grown closely associated with effectiveness, decreased patient injury, and enhanced surgical results, thanks to technology, such as minimally invasive procedures, robotic help, and real-time imaging [30]. The surgeon's skill in maneuvering complex anatomical elements with precision is now a tangible achievement, significantly impacting the patient's recovery after surgery. Precision serves as the crucial link between safety and patient well-being, guaranteeing that therapies are safe and produce the best possible outcomes. The primary objective of surgical efforts is to achieve patient well-being, which goes beyond basic survival and includes a comprehensive perspective on health [31]. An approach that prioritizes the needs and experiences of the patient, characterized by understanding and customized treatment programs, acknowledges the distinctiveness of each person's path. Surgeons recognize the need to prioritize patient well-being by considering the relationship between safety, precision, and overall health outcomes [32]. The holistic approach encompasses the operating room and the surgical journey, including preoperative considerations and thorough aftercare.

The interdependent connection between surgical safety, accuracy, and patient well-being is most apparent in the triumphs resulting from safety and precision enhancements. In several cases, improvements in technology and procedural protocols directly correlate with improved patient outcomes. Minimally invasive procedures in cardiac surgery have brought about a significant transformation in treating heart diseases [33]. Minimally invasive techniques offer patients decreased pain, accelerated recovery periods, and reduced postoperative complications in comparison to standard open-heart surgery. The precise relationship between increased accuracy and better patient results is evidence of the significant impact of advancing surgical techniques [34]. The introduction of modern imaging technologies and navigation systems in neurosurgery has significantly increased the level of precision to an unparalleled extent. Surgeons can precisely map complex brain structures, allowing them to perform focused operations while causing minimum disturbance to the surrounding tissues [35]. The effect on patient outcomes is significant, resulting in decreased neurological impairments, shorter hospitalizations, and enhanced overall quality of life. Neurosurgical precision reduces the chance of causing unintended harm. Moreover, it plays a crucial role in promoting the patient's recovery after the operation [36].

Orthopedic surgery exemplifies the correlation between safety, precision, and patient outcomes. Computer-assisted navigation technologies have been introduced in joint replacement surgeries to provide accurate alignment and ideal positioning of prosthetic components. Such accuracy enhances the durability of joint replacements and results in superior functional outcomes for patients [37]. The meticulous precision provided by technology breakthroughs in orthopedic surgery directly leads to less postoperative discomfort, faster recovery, and enhanced joint function. The comprehensive strategy for achieving successful surgical outcomes is exemplified by these cases in which advancements in safety and accuracy combine to improve patient results [38]. The progression from safety protocols to precision techniques ultimately leads to patient well-being, where the concrete advantages of surgery go beyond the immediate period after the operation [39]. Customized care plans, designed to meet each patient's unique needs, enhance the beneficial effects, highlighting the need to view the patient as a complete entity rather than solely focusing on a particular medical ailment [40].

Ultimately, the intricate relationship between surgical safety, accuracy, and patient well-being establishes the foundation of contemporary surgical procedures. The advancement of safety and precision demonstrates technology development and directly enhances patient outcomes [22]. A complete approach, typified by patient-centered care and thorough assessment of the entire surgical process, emerges as the critical factor in overall surgical success. As we further understand the intricacies of these interwoven elements, the story of exceptional surgical performance appears as a fabric created with the ideals of safety, accuracy, and dedication to the long-term well-being of each patient.

Challenges and future directions

Within the dynamic realm of surgical practice, the constant endeavor for the utmost safety, accuracy, and favorable patient results is characterized by obstacles that stimulate self-reflection and drive the search for enhancement. The present obstacles in attaining these objectives involve complex problems ranging from technology constraints to systemic hindrances and ethical deliberations [23]. As we face these issues, it is crucial to identify possible paths for future research and technological progress while also considering the ethical considerations involved in developing surgical techniques. Attaining the highest level of surgical safety continues to be an ongoing problem despite notable advancements in healthcare [25]. A significant challenge arises from the heterogeneity of individual patient reactions to surgical procedures. Various patient characteristics, including age, comorbidities, and genetic predispositions, contribute to a wide range of responses, which presents difficulties in consistently implementing established safety standards [27]. Moreover, the intricate nature of surgical procedures necessitates a significant degree of coordination among diverse teams, creating the potential for communication failures and human fallibility [29]. To tackle these issues, improving current safety protocols, creating new and inventive methods considering unique patient traits, and enhancing teamwork in the operating room are necessary.

Surgery encounters specific difficulties when it comes to achieving accuracy, primarily because of the constraints imposed by technological limits. Although robotic surgery and real-time imaging have improved precision, these technologies are not widely available. They may still need more complexity for specific procedures [30]. Moreover, acquiring proficiency in new technologies might undermine accuracy in the early stages of adoption. Implementing uniform training programs and promoting cutting-edge technologies' advancement and widespread availability are crucial to addressing these obstacles. Despite ongoing efforts to enhance patient outcomes, some difficulties affect these results beyond the operating room [32]. Postoperative complications, insufficient rehabilitation, and inequalities in accessing follow-up treatment contribute to the diversity in patient outcomes. Socioeconomic factors, such as the availability of healthcare resources and support systems, significantly influence the course of postoperative recovery. To tackle these issues, a complete approach is required to consider the surgical procedure and the broader context of patient care, including preoperative preparation and postoperative rehabilitation [35].

Amid the existing challenges, several possibilities for future study and technical improvements become apparent as we navigate them. Research efforts should concentrate on creating prognostic models that incorporate individualized patient data to anticipate possible consequences and customize interventions accordingly [36]. AI applications have the potential to significantly contribute to analyzing extensive datasets to detect trends and provide individualized treatment strategies. Moreover, the progress in telemedicine and remote monitoring technology can potentially improve postoperative care, particularly for patients in underserved or rural regions, thereby reducing health disparities [36]. The advancements in technology, namely in precision medicine, offer the potential for surpassing existing constraints. Further advances in robotics, augmented reality, and machine learning have the potential to enhance surgical techniques, allowing for increased accuracy and broadening the range of minimally invasive operations [38]. Furthermore, progress in 3D printing and tissue engineering has the potential to transform organ transplants and reconstructive procedures completely. This might lead to personalized implants and tissues being explicitly created for each patient [12].

Nevertheless, as technology continues to evolve, it becomes imperative to address the ethical ramifications arising from developing new surgical techniques. An ethical dilemma arises about the fair allocation of sophisticated technologies. It is crucial to ensure that advanced surgical procedures and technology are available to everyone, regardless of their socioeconomic situation, to avoid worsening healthcare inequalities [13]. Moreover, the conscientious incorporation of AI and automation prompts inquiries regarding the ethical application of these technologies in decision-making and the possibility of depersonalization in patient care. Achieving a harmonious relationship between technical advancement and ethical concerns necessitates continuous discussion, solid regulatory structures, and a dedication to maintaining the principles of patient self-governance and promoting well-being [15]. The ethical ramifications go beyond the scope of access and technology and encompass the areas of informed consent and transparency. With the increasing complexity and technical advancements in surgical procedures, it becomes more challenging to communicate the possible risks and benefits to patients effectively [18]. Surgeons and healthcare providers must carefully convey understandable information to patients while avoiding the unneeded creation of worry [19]. This ensures that patients actively participate in making decisions about their treatment.

The difficulties in attaining the highest level of surgical safety, accuracy, and patient results highlight the intricacy of the healthcare environment. Nevertheless, these challenges also function as catalysts for innovation, propelling the limits of study and technology. Future trends in surgical practice entail a dedication to personalized, patient-focused care, utilizing technological breakthroughs to improve accuracy and safety. Simultaneously, it is crucial to prioritize ethical issues to enhance surgical techniques responsibly. This will ensure that progress is fair, open, and in line with the ideals of patient-centered care.

## Conclusions

The intricate and changing relationship between surgical safety, precision, and patient well-being is a complex topic in healthcare. As we face difficulties in attaining the best results, the significance of a comprehensive strategy becomes evident as the crucial element of surgical success. The quest for safety and precision is closely linked to the larger framework of patient-centered care, which prioritizes every individual's distinct requirements and encounters. The current difficulties highlight the complexities of the surgical process. However, promising opportunities for future study and technological progress suggest a future where surgery becomes more individualized and technologically advanced. Amid these developments, ethical considerations should direct the appropriate development of surgical techniques, guaranteeing that progress is fair, transparent, and in line with the ideals of patient-centered care. As surgical practices advance, the focus on providing personalized, compassionate, and thorough care remains a top priority. This ensures that each patient's well-being is integrated into the core of surgical practice, leading to a prosperous future.
